# Burden and determinants of anaemia among in‐school young adolescents in Ethiopia, Sudan and Tanzania

**DOI:** 10.1111/mcn.13439

**Published:** 2023-03-30

**Authors:** Uttara Partap, Amare W. Tadesse, Sachin Shinde, Huda Sherfi, Isabel Mank, Mary Mwanyika‐Sando, Deepika Sharma, Till Baernighausen, Roisin Drysdale, Alemayehu Worku, Amani Tinkasimile, Wafaie W. Fawzi

**Affiliations:** ^1^ Department of Global Health and Population Harvard T. H. Chan School of Public Health Boston Massachusetts USA; ^2^ Department of Infectious Disease Epidemiology London School of Hygiene and Tropical Medicine London UK; ^3^ Addis Continental Institute of Public Health Addis Ababa Ethiopia; ^4^ Center for Inquiry into Mental Health Pune India; ^5^ School of Health Sciences Ahfad University for Women Omdurman Sudan; ^6^ Heidelberg Institute of Global Health, Faculty of Medicine and University Hospital, Heidelberg University Heidelberg Germany; ^7^ German Institute for Development Evaluation (Deval) Bonn Germany; ^8^ Africa Academy for Public Health Dar es Salaam Tanzania; ^9^ United Nations Children's Fund New York New York USA; ^10^ Africa Health Research Institute KwaZulu‐Natal South Africa; ^11^ DSI‐NRF Centre of Excellence in Human Development University of the Witwatersrand Johannesburg South Africa; ^12^ School of Public Health Addis Ababa University Addis Ababa Ethiopia; ^13^ Department of Epidemiology Harvard T. H. Chan School of Public Health Boston Massachusetts USA; ^14^ Department of Nutrition Harvard T. H. Chan School of Public Health Boston Massachusetts USA

**Keywords:** adolescent, anaemia, diet, food, and nutrition, Ethiopia, hygiene, Sudan, Tanzania

## Abstract

Anaemia among adolescents is a global health problem. However, evidence regarding its burden and risk factors, particularly for younger adolescents and in sub‐Saharan Africa (SSA), remains scarce. We aimed to assess the prevalence and potential determinants of anaemia among urban and semi‐urban in‐school young adolescents in Ethiopia, Sudan and Tanzania. We conducted a school‐based survey among 3558 adolescents aged 10−14 years. A capillary blood sample was used to assess haemoglobin concentration. We assessed anaemia prevalence and examined associations between measures at the individual, household and school levels and anaemia using Poisson regression models adjusted for school and country‐level clustering. The prevalence of anaemia was 32.0% overall, and 10.8% in Ethiopia, 25.0% in Sudan and 58.3% in Tanzania. Being a boy [adjusted risk ratio (RR): 1.11, 95% confidence interval (CI): 1.08−1.15, *p* < 0.001], poorer diet quality (RR: 1.12, 95% CI: 1.02−1.23 *p* = 0.015), no school handwashing stations (RR: 1.26, 95% CI: 1.20−1.32, *p* < 0.001) and food insecurity (RR for moderate/severe anaemia: 1.06, 95% CI: 1.02−1.10, *p* = 0.002) were associated with increased anaemia risk. Younger age (RR: 0.91, 95% CI: 0.86−0.96, *p* < 0.001) and increasing height‐for‐age *z*‐score (RR: 0.93, 95% CI: 0.91−0.95, *p* < 0.001) were associated with decreased anaemia risk. Associations were consistent for moderate or severe anaemia. There was no evidence of effect modification by sex. This study highlights anaemia as a public health problem and identified nutritional, dietary and hygiene measures as key risk factors of anaemia among young adolescents in SSA. School‐based interventions addressing these factors could reduce the burden of anaemia in adolescence.

## Key messages

- We found a substantial burden of anaemia among urban and semi‐urban in‐school young adolescents living in three major cities in Ethiopia, Sudan and Tanzania.
- The prevalence and adjusted risk of anaemia were higher among boys compared with girls.
- Anaemia risk among young adolescents was associated with multiple modifiable indices, including poor diet quality, food insecurity and unavailability of school handwashing stations.
- These findings indicate a need to address anaemia among both young adolescent boys and girls in this region and point towards targetable potential determinants.
- Schools may serve as valuable platforms for the delivery of interventions targeting anaemia in this age group.

## Graphical abstract

‐ We found a substantial burden of anaemia among urban in‐school young adolescents in Ethiopia, Sudan and Tanzania.

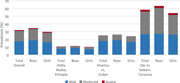

## 1 INTRODUCTION

Anaemia is a common blood disorder. The age‐standardized global prevalence of anaemia was estimated to be 22.8% in 2019, with approximately 1.74 billion people affected, and it was associated with 58.6 (41.0−81.1) million years lost to disability that year (Gardner & Kassebaum, 2020). Anaemia is defined by low haemoglobin levels relative to age, sex and pregnancy status, and is known to have a range of potential causes, including nutritional deficiencies (such as iron and folate deficiency), blood loss, hemoglobinopathies and other conditions affecting haemoglobin synthesis and erythrocyte production and survival, and other infectious or chronic causes such as malaria or chronic kidney disease (Kassebaum, 2016). It is understood to contribute to a number of poor health outcomes, including adverse pregnancy outcomes, impaired child motor and mental development and reduced work productivity among others (Kassebaum, 2016). Given the global scale of the burden of anaemia, further work is required to find effective solutions to address it.

Much of the previous literature and programmatic action on anaemia has focused on pregnant women and young children, due to evidence of its disproportionate prevalence in these groups (Kassebaum, 2016). However, recent data point towards a notable burden of anaemia among adolescents aged 10−19 years, with up to 25%−30% of adolescents affected in lower social development index settings (Christian & Smith, 2018; Guthold et al., 2021; Kassebaum, 2016). It is understood that adolescents may be particularly vulnerable to anaemia because of changes in physiology and related increases in nutritional requirements during this age (Patton et al., 2016). Adolescence is also recognized as a key period to influence the adoption of healthy habits that may affect nutrition and health outcomes (including anaemia) throughout life and across generations, such as dietary practices (Frech, 2012; Mikkilä et al., 2005; Patton et al., 2016). Anaemia among adolescents is particularly important to address given its impact on outcomes including the ability to concentrate, fatigue and adverse pregnancy outcomes (Chaparro & Suchdev, 2019; Kassebaum, 2016; World Health Organization, 2017). Comprehensive evidence regarding anaemia during adolescence is therefore warranted to better understand potentially important contemporary and future implications for this age group.

Despite this, there are limited data on the burden of and specific risk factors for anaemia throughout adolescence, especially regarding young adolescents from 10 to 14 years of age (Christian & Smith, 2018). These may be distinct from risk factors among other population groups, such as adults. Furthermore, there is very little evidence from sub‐Saharan Africa (SSA), despite the fact that the region has a large number of adolescents (249 million out of 1.2 billion globally in 2019), and adolescents comprise the greatest proportion of the population in the region (UNICEF, 2019). A clearer understanding of anaemia among adolescents in SSA would help inform appropriate responses for prevention and management in the region. We, therefore, sought to understand the prevalence of and potential risk factors for anaemia among urban and semi‐urban school‐going adolescents aged 10−14 years living in three major cities in Ethiopia, Sudan and Tanzania.

## 2 METHODS

### 2.1 Study setting and design

We conducted a school‐based cross‐sectional study in cities of three sub‐Saharan African countries: Ethiopia (Addis Ababa), Sudan (Khartoum) and Tanzania (Dar es Salaam). The population of Addis Ababa was estimated to be 4.59 million in 2019 (UNData, 2022b), while the population of Khartoum was estimated at 5.68 million in 2019 (UNData, 2022c), and the population of Dar es Salaam was approximately 4.36 million in 2012 (UNData, 2022a). At the time of sampling, there were 221 government schools in Addis Ababa, 1567 in Khartoum and 327 in Dar es Salaam.

Briefly, the sampling and study procedures are as follows. We used multistage sampling with the aim of selecting approximately 1200 students aged 10−14 at each site. The number of administrative units sampled per city was based on feasibility: we included all 10 subcities in Addis Ababa and all 5 administrative districts in Dar es Salaam, and randomly sampled 4 out of 7 districts in Khartoum. Within each administrative unit, we randomly sampled 2−4 schools. Within each school, we performed stratified random sampling by grade, randomly selecting 15−25 students from each of the 4 relevant grades for a total of approximately 60−100 students per school.

Students were administered a questionnaire in face‐to‐face interviews with trained data collectors. The questionnaire was pre‐programmed into electronic devices, and was based on the WHO Global School‐based Student Health Survey (WHO, 2021) with additional sections from other validated instruments covering key individual and household questions. These questions included demographic, health, dietary, water, sanitation and hygiene (WASH), physical activity, socioemotional and educational information. Height and weight measurements were then taken in duplicate following standard procedures (WHO, 2006) and using UNICEF SECA scales and height measuring boards. The average height and weight measurements were calculated from the two values. Finally, haemoglobin was measured with a portable HemoCue machine (HemoCue 201) using a capillary blood sample collected from the fingertip of each participant using an aseptic technique through sterile single‐use disposable lancet and 70% ethanol alcohol disinfectant. Blood sample collection and testing were carried out by trained and experienced laboratory technicians. All safety measures were taken during blood sample collection.

Teachers were interviewed to gather information relating to health, nutrition and WASH practices and policies at the school. Information collected included items such as the existence of school policies, strategies or guidelines related to health and nutrition and the availability of specific resources and services including school feeding, toilets and counselling.

### 2.2 Variable definitions

Haemoglobin levels were adjusted by altitude in Ethiopia due to the altitude of study sites in this country being above 1000 m, as recommended previously (Sullivan et al., 2008). Anaemia was defined in accordance with World Health Organization (WHO) guidelines, using a haemoglobin cut‐off value of <11.5 g/dl for adolescents aged between 10 and 11 years and <12 g/dl for those between 12 and 14 years. We further classified mild, moderate or severe anaemia as 11.0−11.4, 8.0−10.9 and <8.0 g/dl among adolescents aged 10−11 years, and 11.0−11.9, 8.0−10.9 and <8.0 g/dl among those between 12 and 14 years (WHO, 2011). As binary outcomes, we examined any versus no anaemia, and moderate or severe versus mild or no anaemia. In sensitivity analyses, we also considered moderate or severe versus no anaemia as an outcome to assess the consistency of associations.

Based on previous research on factors that were associated with anaemia among adolescents (Azupogo et al., 2019; Engidaw et al., 2018; Ford et al., 2020; Gosdin et al., 2020; Korkalo et al., 2017; Regasa & Haidar, 2019; Shaka & Wondimagegne, 2018), we considered the following variables as potential risk factors: age, sex, height‐for‐age *z*‐score (HAZ), diet quality and dietary diversity score, living with both parents, whether menarche had been reached (for girls only), maternal education, household asset index, household food insecurity, handwashing after going to the toilet, school feeding programmes, available dietary guidelines at school and available handwashing stations at school.

Food consumption was assessed using a 24 h recall and a structured 7‐day food frequency questionnaire. Dietary diversity was assessed based on 24 h recall and using guidance provided by the Food and Agriculture Organization, to compute the minimum dietary diversity for women of reproductive age (MDD‐W) indicator (score range: 0−10, poor dietary diversity defined as score <5) (USAID & FAO, 2016). This is based on 10 food groups: starchy staples; beans and peas; nuts and seeds; dairy; flesh foods (meat, fish); eggs; vitamin A dark green leafy vegetables; other vitamin A rich fruits and vegetables; other vegetables and other fruits.

To assess diet quality, foods consumed by adolescents 7 days before the survey were classified into 25 food groups to calculate global diet quality score (GDQS; score range: 0−49), and 21 food groups to calculate prime diet quality score (PDQS; score range: 0−42). The GDQS is a recently‐developed metric for the assessment of diet quality aimed for use in population‐based surveys globally (Intake, 2021). For the GDQS, food groups were defined and classified as healthy, unhealthy or unhealthy when consumed in excessive amounts, in accordance with guidelines provided by Intake—Center for Dietary Assessment (Intake, 2021). The only exception in classification was high‐fat dairy, which is classified as unhealthy in excessive amounts as per Intake, but was categorized as healthy in this analysis. This was done as we observed a low to moderate frequency of dairy consumption per week. Points for low, middle, high or very high intake for each food group were assigned based on the amount consumed (reported frequency) and summed, with higher overall scores corresponding to healthier diet quality. Country‐specific median points were used for individuals who had data missing on intake of specific food groups (<2.6% of observations for any variable). GDQS <15 and ≥23 were defined as low and high diet quality respectively, in accordance with recommended population‐based cutoffs (Intake, 2021). For the PDQS, points were assigned for consumption of healthy food groups as 0−1 serving/week (0 points), 2−4 servings/week (1 point) and ≥5 servings/week (2 points). Scoring for unhealthy food groups was assigned as 0−1 serving/week (2 points), 2−4 servings/week (1 point) and ≥5 servings/week (0 points). Points for each food group were then summed to give an overall score (Fung et al., 2018; Rifas‐Shiman et al., 2001), and quartiles were created for analytical purposes.

Food insecurity was measured using the household hunger scale developed by the Food and Nutrition Technical Assistance III Project (score range: 0−6) (Ballard et al., 2011). HAZ was calculated using the WHO AnthroPlus software, and stunting was defined as HAZ <−2, in accordance with WHO guidelines (de Onis et al., 2007; WHO, 2014a, 2014b).

Household asset index was calculated by country, based on ownership of assets (electricity, radio, television, mobile phone, refrigerator, washing machine, computer, photo camera, DVD or CD player, bed or mattress, table, chair, cabinet or cupboard, bicycle, motorcycle or scooter, car or truck, solar panel) using principal component analysis. Maternal education was classified to reflect categories of literacy and educational level. School‐level measures collected from teachers' interviews (school feeding, dietary guidelines at school and handwashing stations at school) were included as binary variables, and not transformed further.

### 2.3 Statistical analysis

We first examined the crude prevalence of anaemia (any anaemia, and anaemia split by severity) overall and by sex and country. We also examined the distribution of any anaemia across variables of interest; differences across categories were assessed using Pearson's *χ* ^2^ test or Fisher's exact test for comparisons having cell counts <5.

Following this, generalized linear models (Poisson link and robust standard error) were used to evaluate univariable associations between variables of interest and anaemia in adolescents, pooled across countries. The missing indicator method was used to account for missingness in the maternal education variable (information on all other variables was generally complete). Variables with *p* < 0.2 in the unadjusted models were then fitted in multivariable models, which were adjusted for clustering at the school and country levels. For diet quality, only GDQS was included in the main models, while PDQS was examined in sensitivity analyses to check differences in associations by the measure used (see below).

Main multivariable models considered any anaemia (vs. no anaemia) as an outcome. Furthermore, to assess potential differences in the impact of potential risk factors across anaemia severity, we additionally considered moderate or severe anaemia (vs. no or mild anaemia) as an outcome. We tested the proportionality of odds across categories of anaemia (no, mild, moderate or severe) by running fully adjusted ordinal logistic regression models followed by the Brant test. The *p* value for the Brant test was 0.008, indicating a violation of the proportional odds assumption and supporting the examination of anaemia categories in separate models. Following this, given previous literature suggesting differences in the effects of risk factors across boys and girls (Azupogo et al., 2019; Gosdin et al., 2020), we stratified models by sex. For girls, we built and checked multivariable models both including and excluding menarche as a covariate. As menarche was not associated with anaemia risk in these fully‐adjusted models and did not substantially affect other associations, models excluding menarche (i.e., models identically adjusted to those for boys) were used for girls. Effect modification of each potential risk factor by sex was assessed using likelihood ratio tests comparing main multivariable models nested within the same models additionally including relevant interaction terms.

Finally, we conducted a number of sensitivity analyses to assess the robustness of results, including: (1) examining associations for moderate or severe anaemia versus no anaemia as an outcome (i.e., excluding mild cases from the reference group), (2) assessing associations for haemoglobin as an outcome in mixed effects multivariable linear regression models identically adjusted to main models, (3) using PDQS instead of GDQS as an indicator of diet quality. Additionally, we also examined estimates obtained when considering all possible variables in models adjusted cumulatively for sociodemographic, school‐related, dietary and hygiene practice‐related and anthropometric variables (Model 1: adjusted for sex, age, living with both parents, maternal highest education and household asset index; Model 2: Model 1 plus school feeding, dietary guidelines and handwashing stations; Model 3: Model 2 plus food insecurity, dietary diversity, diet quality and washing hands after going to the toilet; Model 4: Model 3 plus HAZ). This is considered a robust method of estimating effect that takes into account the hierarchical nature of variables (Victora et al., 1997). Analyses were conducted using Stata 16 (StataCorp).

### 2.4 Ethical consideration

Ethical approval was received from the institutional ethical review board of the Addis Continental Institute of Public Health, Ethiopia; Ahfad University for Women and the Ministries of Education and Health, Sudan; the National Institute for Medical Research, Tanzania; and the Harvard T. H. Chan School of Public Health Institutional Review Board. Permission to conduct the study was obtained from regional and district education health offices, and school administration. The Declaration of Helsinki was followed when conducting the study. For the adolescent survey, informed written consent from parents or guardians of adolescents and assent from adolescents were obtained. For the school survey, written consent was obtained from teachers and school principals.

## 3 RESULTS

Out of a total of 3558 surveyed adolescents, analyses covered a total of 3554 adolescents with information on haemoglobin (99.9%) (Table 1). Approximately half of participants were girls [*N* = 1864 (52.4%)], and participants were almost evenly distributed across the three settings [Addis Ababa, Ethiopia: *N* = 1200 (33.8%); Khartoum, Sudan: *N* = 1099 (30.9%); Dar es Salaam, Tanzania: *N* = 1255 (35.3%)]. Over half of the participants were aged 10−12 years [*N* = 2125 (59.8%)]. Most participants lived with both parents [*N* = 2428 (68.3%)]. Of participants with available information, over half had mothers with secondary education or higher [*N* = 1366/2581 (52.9%)]. Approximately one‐tenth of participants overall were stunted [*N* = 316 (8.9%)], with slight variations in prevalence across cities. Dietary diversity was calculated to be poor for most adolescents [*N* = 2828 (79.6%)], and the majority of participants had moderate or low diet quality as assessed by the GDQS [*N* = 2482 (69.8%)]. A small proportion of adolescents reported some level of household food insecurity [moderate or severe hunger: *N* = 216 (6.1%)]. Approximately half of all adolescents had access to school feeding programmes [*N* = 1695 (47.7%)] and three‐quarters had access to handwashing stations at school [*N* = 2666 (75.0%)]. Fewer adolescents went to schools which had dietary guidelines [*N* = 481 (13.5%)] (Table 1).

**Table 1:** Individual, household and school characteristics of in‐school adolescents aged 10−14 years by anaemia status in Addis Ababa (Ethiopia), Khartoum (Sudan) and Dar es Salaam (Tanzania)

	Anaemia, *n*/*N* a (%)			
	Overall	Addis Ababa, Ethiopia	Khartoum, Sudan	Dar es Salaam, Tanzania
Overall, *N*	1136/3554 (32.0%)	129/1200 (10.8%)	275/1099 (25.0%)	732/1255 (58.3%)
Sex of participant, *N*	3554	1200	1099	1255
Male	583/1690 (34.5)	62/543 (11.4)	143/548 (26.1)	378/599 (63.1)
Female	553/1864 (29.7)	67/657 (10.2)	132/551 (24.0)	354/656 (54.0)
*p*	0.002	0.497	0.413	0.001
Age of participant (years), *N*	3554	1200	1099	1255
10−12	736/2125 (34.6)	51/536 (9.5)	151/642 (23.5)	534/947 (56.4)
13−14	400/1429 (28.0)	78/664 (11.7)	124/457 (27.1)	198/308 (64.3)
*p*	<0.001	0.215	0.173	0.015
Menarche (girls only)	1864	657	551	656
Yes	146/532 (27.4)	34/289 (11.8)	36/124 (29.0)	76/119 (63.9)
No	407/1330 (30.6)	33/368 (9.0)	96/425 (22.6)	278/537 (51.8)
Missing	0/2 (0.0)	0/0 (−)	0/2 (0.0)	0/0 (−)
*p*	0.276	0.240	0.229	0.017
Live with both parents	3554	1200	1099	1255
Yes	743/2428 (30.6)	72/689 (10.4)	257/1015 (25.3)	414/724 (57.2)
No	393/1126 (34.9)	57/511 (11.2)	18/84 (21.4)	318/531 (59.9)
*p*	0.011	0.697	0.429	0.337
Wash hands after toilet, *N*	3554	1200	1099	1255
Rare/sometimes	584/1626 (35.9)	26/217 (12.0)	143/693 (20.6)	415/716 (58.0)
Mostly/always	552/1927 (28.6)	103/982 (10.5)	132/406 (32.5)	317/539 (58.8)
Missing	0/1 (0)	0/1 (0)	0/0 (0)	0/0 (0)
*p*	<0.001	0.521	<0.001	0.762
Maternal highest education, *N*	3554	1200	1099	1255
Primary or less	376/1215 (30.9)	76/655 (11.6)	13/66 (19.7)	287/494 (58.1)
Secondary or above	349/1366 (25.5)	30/318 (9.4)	196/816 (24.0)	123/232 (53.0)
Missing	411/973 (42.2)	23/227 (10.1)	66/217 (30.4)	322/529 (60.9)
*p*	<0.001	0.560	0.091	0.128
Household asset index, *N*	3554	1200	1099	1255
Lowest quintile	247/714 (34.6)	21/232 (9.1)	66/230 (28.7)	160/252 (63.5)
Low quintile	223/694 (32.1)	21/232 (9.1)	49/211 (23.2)	153/251 (61.0)
Middle quintile	211/703 (30.0)	30/264 (11.4)	49/187 (26.2)	132/252 (52.4)
High quintile	222/729 (30.5)	25/232 (10.8)	54/248 (21.8)	143/249 (57.4)
Highest quintile	233/714 (32.6)	32/240 (13.3)	57/223 (25.6)	144/251 (57.4)
*p*	0.352	0.530	0.465	0.120
Height‐for‐age category, *N*	3554	1200	1099	1255
Stunted	130/316 (41.1)	11/98 (11.2)	15/60 (25.0)	104/158 (65.8)
Not stunted	1006/3238 (31.1)	118/1102 (10.7)	260/1039 (25.0)	628/1097 (57.2)
*p*	<0.001	0.874	0.997	0.041
Dietary diversity (MDD‐W), *N*	3554	1200	1099	1255
Poor	983/2828 (34.8)	74/756 (9.8)	223/896 (24.9)	686/1176 (58.3)
Adequate	153/726 (21.1)	55/444 (12.4)	52/203 (25.6)	46/79 (58.2)
*p*	<0.001	0.161	0.829	0.985
Prime diet quality score (PDQS)*, N*	3554	1200	1099	1255
4th (highest) quartile	208/763 (27.3)	14/150 (9.3)	87/420 (20.7)	107/193 (55.4)
3rd quartile	223/852 (26.2)	40/324 (12.3)	76/321 (23.7)	107/207 (51.7)
2nd quartile	324/989 (32.8)	47/433 (10.9)	78/231 (33.8)	199/325 (61.2)
1st (lowest) quartile	381/950 (40.1)	28/293 (9.6)	34/127 (26.8)	319/530 (60.2)
*p*	<0.001	0.656	0.003	0.098
Global diet quality score (GDQS)*, N*	3554	1200	1099	1255
High	300/1072 (28.0)	21/181 (11.6)	143/652 (21.9)	136/239 (56.9)
Moderate	724/2249 (32.2)	107/983 (10.9)	129/442 (29.2)	488/824 (59.2)
Low	112/233 (48.1)	1/36 (2.8)	3/5 (60.0)	108/192 (56.3)
*p*	<0.001	0.298	0.004	0.666
Food insecurity (HHS), *N*	3554	1200	1099	1255
Little/no hunger	1035/3338 (31.0)	125/1158 (10.8)	267/1070 (25.0)	643/1110 (57.9)
Moderate hunger	87/193 (45.1)	4/40 (10.0)	8/29 (27.6)	75/124 (60.5)
Severe hunger	14/23 (60.9)	0/2 (0.0)	0/0 (0.0)	14/21 (66.7)
*p*	<0.001	1.000	0.828	0.634
School feeding, *N*	3554	1200	1099	1255
Yes	388/1695 (22.9)	117/1080 (10.8)	65/315 (20.6)	206/300 (68.7)
No	748/1859 (40.2)	12/120 (10.0)	210/784 (26.8)	526/955 (55.1)
*p*	<0.001	0.780	0.033	<0.001
School dietary guidelines, *N*	3554	1200	1099	1255
Yes	69/481 (14.3)	46/360 (12.8)	23/121 (19.0)	732/1255 (58.3)
No	1067/3073 (34.7)	83/840 (9.9)	252/978 (25.8)	0/0 (0.0)
*p*	<0.001	0.138	0.105	‐
School handwashing stations, *N*	3554	1200	1099	1255
Yes	804/2666 (30.2)	124/1140 (10.9)	118/504 (23.4)	562/1022 (55.0)
No	332/888 (37.4)	5/60 (8.3)	157/595 (26.4)	170/233 (73.0)
*p*	<0.001	0.535	0.257	<0.001

*Note*: *p* values based on *χ* ^2^ tests (Fisher's exact tests for variables with cell counts <5).

Abbreviations: HHS, household hunger scale score; MDD‐W, minimum dietary diversity for women of reproductive age.

^a^ *n*/*N*, number of individuals with anaemia (*n*)/total number of individuals within the category (*N*).

**Figure 1:**
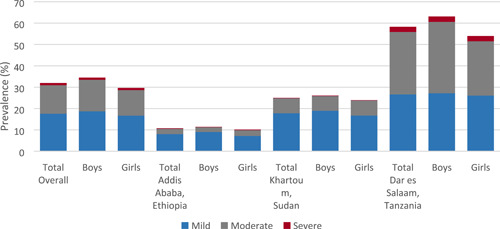
Prevalence of anaemia among adolescents in Addis Ababa (Ethiopia), Khartoum (Sudan) and Dar es Salaam (Tanzania)

The overall prevalence of anaemia in the study population was 32.0%. Notable differences could be observed across settings, with the lowest prevalence in Addis Ababa (10.8%), and the highest in Dar es Salaam (58.3%) (Figure 1, Table 1). Mild anaemia accounted for the greatest proportion of cases in Addis Ababa and in Khartoum, whereas there was a more even distribution between mild and moderate anaemia in Dar es Salaam (prevalence of mild anaemia: 26.6%, of moderate anaemia: 29.2%) (Figure 1). Anaemia prevalence was marginally higher among boys than girls, with similar distributions by severity between the two groups overall and across cities (Table 1, Figure 1).

There was some evidence of decreased anaemia prevalence with increasing maternal education and among adolescents reporting mostly washing hands after going to the toilet (*p* < 0.001 for both). On the other hand, anaemia prevalence was higher among stunted individuals, those with lower diet quality and higher household hunger scale scores, and those who did not have access to school feeding, dietary guidelines at school or handwashing stations at school (*p* < 0.001 for all) (Table 1). However, trends were generally not fully consistent across cities (Table 1).

In multivariable analysis, increased risk of any anaemia was associated with being a boy [risk ratio (RR): 1.11, 95% confidence interval (95% CI): 1.08−1.15, *p* < 0.001], having moderate or low diet quality (RR for moderate vs. high GDQS: 1.12, 95% CI: 1.02−1.23, *p* = 0.015, *p* for trend = 0.175), and not having handwashing stations at school (RR: 1.26, 95% CI: 1.20−1.32, *p* < 0.001) (Table 2). Being of younger age was associated with a decreased risk of anaemia (RR for 10−12 vs. 13−14 years: 0.91, 95% CI: 0.86−0.96, *p* < 0.001). HAZ was similarly associated with reduced risk (RR per unit increase in HAZ: 0.93, 95% CI: 0.91−0.95, *p* < 0.001) (Table 2).

**Table 2:** Associations of individual, household and school characteristics with anaemia among adolescents in Addis Ababa (Ethiopia), Khartoum (Sudan) and Dar es Salaam (Tanzania)

	Any anaemia		Moderate or severe anaemia^a^	
	(*N* = 3553)		(*N* = 3553)	
	RR (95% CI)	*p*	RR (95% CI)	*p*
Sex of participant				
Male	1.11 (1.08−1.15)	<0.001	1.16 (1.04−1.29)	0.006
Female	Ref		Ref	
Age of participant (years)				
10–12	0.91 (0.86−0.96)	<0.001	1.05 (0.95−1.17)	0.322
13–14	Ref		Ref	
Height‐for‐age *z*‐score	0.93 (0.91−0.95)	<0.001	0.92 (0.90−0.95)	<0.001
Live with both parents				
No	1.02 (1.00**−**1.05)	0.103	1.02 (0.90**−**1.15)	0.768
Yes	Ref		Ref	
Wash hands after toilet				
Rare/sometimes	0.90 (0.75**−**1.07)	0.237	0.92 (0.82**−**1.02)	0.123
Mostly/always	Ref		Ref	
Maternal highest education				
Primary or less	1.08 (0.97**−**1.20)	0.183	1.09 (0.90**−**1.33)	0.367
Secondary or above	Ref		Ref	
Dietary diversity (MDD‐W)				
Poor	0.93 (0.81**−**1.07)	0.314	0.90 (0.55**−**1.49)	0.691
Adequate	Ref		Ref	
Global diet quality score (GDQS)				
High	Ref		Ref	
Moderate	1.12 (1.02−1.23)	0.015	1.14 (1.10−1.17)	<0.001
Low	1.09 (0.92−1.30)	0.307	1.20 (1.12−1.28)	<0.001
*p* for linear trend		0.175		<0.001
Food insecurity (HHS)	1.00 (0.99−1.01)	0.791	1.06 (1.02−1.10)	0.002
School feeding				
No	1.00 (0.69**−**1.43)	0.983	0.84 (0.49**−**1.43)	0.518
Yes	Ref		Ref	
School dietary guidelines				
No	0.91 (0.65**−**1.26)	0.568	0.95 (0.52**−**1.72)	0.865
Yes	Ref		Ref	
School handwashing stations				
No	1.26 (1.20−1.32)	<0.001	1.37 (0.93**−**2.01)	0.107
Yes	Ref		Ref	

*Note*: Estimates based on Poisson regression models adjusting for all other variables displayed, and for clustering at the school and country level.

Abbreviations: 95% CI, 95% confidence interval; HHS, household hunger scale score; MDD‐W, minimum dietary diversity for women of reproductive age; RR, risk ratio.

^a^ Comparator: no or mild anaemia.

When examining moderate or severe anaemia as an outcome (vs. no or mild anaemia), associations remained largely consistent in terms of effect size and direction, although the effect of age and not having access to handwashing stations at school were no longer statistically significant. Additionally, food insecurity was associated with 6% increased risk of moderate or severe anaemia (RR per unit increase in household hunger scale score: 1.06, 95% CI: 1.02−1.10, *p* = 0.002) (Table 2).

We further explored potential effect modification by sex (Table 3). In stratified models, associations between potential determinants and any anaemia were generally similar across boys and girls, with overlapping CIs and no clear statistical evidence of interaction when assessed using likelihood ratio tests (Table 3).

**Table 3:** Associations of individual, household and school characteristics with anaemia among adolescents in Addis Ababa (Ethiopia), Khartoum (Sudan) and Dar es Salaam (Tanzania); stratification by sex

	Any anaemia					Moderate or severe anaemia^a^				
	Boys		Girls			Boys		Girls		
	(*N* = 1690) RR (95% CI)	*p*	(*N* = 1863) RR (95% CI)	*p*	*p* for interaction	(*N* = 1690) RR (95% CI)	*p*	(*N* = 1863) RR (95% CI)	*p*	*p* for interaction
Age of respondent (years)										
10–12	1.01 (0.85−1.20)	0.896	0.76 (0.66−0.89)	<0.001	0.092	1.29 (1.10−1.51)	0.002	0.77 (0.71−0.84)	<0.001	0.022
13–14	Ref		Ref			Ref		Ref		
Height‐for‐age *z*‐score	0.91 (0.85−0.98)	0.014	0.98 (0.95−1.00)	0.056	0.450	0.90 (0.84−0.96)	0.002	1.01 (0.88−1.16)	0.880	0.325
Live with both parents										
No	1.04 (1.01−1.08)	0.023	0.99 (0.99−1.00)	0.003	0.806	1.08 (1.06−1.10)	<0.001	0.93 (0.73−1.18)	0.549	0.612
Yes	Ref		Ref			Ref		Ref		
Wash hands after toilet										
Rare/sometimes	0.92 (0.82−1.02)	0.127	0.88 (0.66−1.18)	0.401	0.673	1.05 (0.97−1.14)	0.219	0.80 (0.66−0.98)	0.029	0.125
Mostly/always	Ref		Ref			Ref		Ref		
Maternal highest education										
Primary or less	1.14 (0.89−1.47)	0.301	1.02 (0.88−1.19)	0.778	0.328	1.13 (1.04−1.23)	0.005	1.12 (0.87−1.42)	0.377	0.981
Secondary or above	Ref		Ref			Ref		Ref		
Dietary diversity (MDD‐W)										
Poor	0.91 (0.83−1.00)	0.041	0.97 (0.80−1.18)	0.762	0.814	0.93 (0.57−1.53)	0.778	0.92 (0.55−1.56)	0.760	0.628
Adequate	Ref		Ref			Ref		Ref		
Global diet quality score										
High	Ref		Ref			Ref		Ref		
Moderate	1.14 (1.01−1.30)	0.034	1.09 (0.98−1.20)	0.097	0.264	1.25 (1.22−1.28)	<0.001	1.02 (0.94−1.11)	0.590	0.331
Low	1.32 (1.15−1.53)	<0.001	0.90 (0.73−1.10)	0.297		1.38 (1.14−1.67)	<0.001	0.99 (0.89−1.10)	0.808	
*p* for linear trend		0.004		0.959			<0.001		0.657	
Food insecurity (HHS)	1.00 (0.98, 1.01)	0.546	1.01 (0.96−1.05)	0.814	0.958	1.07 (1.00−1.13)	0.036	1.04 (1.02−1.05)	<0.001	0.689
School feeding										
No	1.06 (0.65−1.72)	0.818	0.87 (0.78−0.97)	0.011	0.242	0.85 (0.52−1.40)	0.535	0.71 (0.55−0.91)	0.007	0.187
Yes	Ref		Ref			Ref		Ref		
School dietary guidelines										
No	0.99 (0.71−1.40)	0.972	0.91 (0.59−1.40)	0.659	0.862	1.30 (1.21−1.39)	<0.001	0.87 (0.30−2.54)	0.793	0.223
Yes	Ref		Ref			Ref		Ref		
School handwashing stations										
No	1.18 (1.03−1.34)	0.015	1.42 (1.26−1.59)	<0.001	0.179	1.32 (1.05−1.67)	0.018	1.63 (1.27−2.10)	<0.001	0.312
Yes	Ref		Ref			Ref		Ref		

*Note*: Estimates based on Poisson regression models adjusting for all other variables displayed, and for clustering at the school and country level.

Abbreviations: 95% CI, 95% confidence interval; HHS, household hunger scale score; MDD‐W, minimum dietary diversity for women of reproductive age; RR, risk ratio.

^a^ Comparator: no or mild anaemia.

In sex‐stratified models examining moderate or severe anaemia as an outcome (vs. no or mild anaemia), there was some evidence of modification by sex of the effect of age—with increased risk of anaemia among younger boys but decreased risk among younger girls (10−12 vs. 13−14 years—RR for boys: 1.29, 95% CI: 1.10−1.51, *p* = 0.002; RR for girls: 0.77, 95% CI: 0.71−0.84, *p* < 0.001; *p* for interaction = 0.022). Although there was some evidence of divergence of effects for diet quality, this was not statistically notable (Table 3).

In sensitivity analyses, we observed similar patterns of associations in models assessing moderate or severe anaemia as an outcome regardless of the inclusion of mild cases in the reference group (Supporting Information: Table 1). Associations also remained generally consistent when examining haemoglobin as a continuous outcome in linear regression models (Supporting Information: Table 1), and when using PDQS to assess diet quality (Supporting Information: Table 2). Finally, there were no substantial differences in associations when cumulatively adjusting for sociodemographic factors, school characteristics, dietary and hygiene‐related practices and anthropometric factors (Supporting Information: Table 3).

## 4 DISCUSSION

In this study based among urban and semi‐urban school‐going adolescents in Ethiopia, Sudan and Tanzania, we observed a high overall prevalence of anaemia, with close to one‐third of adolescents affected. Importantly, there were notable differences across the three countries in anaemia prevalence and distribution by severity, with the highest prevalence and a larger proportion of moderate cases in Tanzania. The risk of any anaemia was associated with individual, household and school‐level factors across demographic, anthropometric, dietary and hygiene domains. Associations remained consistent when examining moderate or severe anaemia as an outcome. While there was some indication of differences in the effects of certain factors by sex, statistical evidence of interaction was weak. Together, these findings highlight a notable burden of anaemia among adolescents in these populations and provide information regarding potentially relevant targetable factors and approaches to reduce this burden.

To our knowledge, comprehensive and up‐to‐date data regarding the prevalence of anaemia among adolescents especially within the SSA region remain scarce. Previous studies have included limited reports based on data from Demographic and Health Surveys or similar country‐wide surveys measuring haemoglobin only among adolescent girls aged 15−19 years (ICF, 2021; Mchiza et al., 2018). We found few additional studies published in the past decade examining the anaemia burden among adolescents in any SSA country (Azupogo et al., 2019; Engidaw et al., 2018; Gosdin et al., 2020; Regasa & Haidar, 2019; Seyoum et al., 2019; Shaka & Wondimagegne, 2018). Many of these studies were based among in‐school adolescents (Azupogo et al., 2019; Gosdin et al., 2020; Seyoum et al., 2019; Shaka & Wondimagegne, 2018), with some again based exclusively among older adolescent girls mainly aged 14−19 years (Engidaw et al., 2018; Regasa & Haidar, 2019; Seyoum et al., 2019). Such studies have reported a range of prevalence estimates, from approximately 8.7% among 15−19 year‐old girls in Ethiopia (Seyoum et al., 2019) to 54.6% among boys aged 10−17 in Ghana (Azupogo et al., 2019). Some have highlighted notable within‐country differences across physical geography and rural‐urban setting (Azupogo et al., 2019; Regasa & Haidar, 2019; Shaka & Wondimagegne, 2018). With data from adolescents aged 10−14 years, our study supports and further extends these observations. Our study found a high overall prevalence of anaemia, highlighting the need for a stronger focus on monitoring and addressing anaemia status among younger adolescents in this region. Furthermore, it reinforces country‐specific differences in anaemia burden, suggesting it as a mild public health problem within our study settings in Ethiopia, moderate in Sudan and severe in Tanzania (De Benoist et al., 2008). This indicates potential implications for differences in the intensity of approaches to address anaemia among adolescents within these specific populations.

Similar to our study, previous research has suggested important influences of demographic factors on adolescents' risk of anaemia. Consistent with our observations, certain studies reported positive associations between anaemia risk and age (Engidaw et al., 2018; Gosdin et al., 2020; Shaka & Wondimagegne, 2018). This may be explained by changes in physiology and therefore iron requirements related to growth (Christian & Smith, 2018; Kassebaum, 2016; WHO SEARO, 2011). Furthermore, similar to our findings, two other studies reported a higher prevalence of anaemia among adolescent boys compared with girls aged 10−19 years in Ghana and Ethiopia (Azupogo et al., 2019; Shaka & Wondimagegne, 2018). Previous global data suggest a generally higher prevalence of anaemia among adolescent girls, understood to be due to increased iron requirements following the onset of menarche in this period (Christian & Smith, 2018; Kassebaum, 2016). A notable proportion of girls in this population had not reached menarche, which may to some extent explain our observations. However, menarche was not associated with anaemia risk in our analyses. The current observations thus suggest other potentially important underlying differences by sex that may additionally contribute to differences in anaemia risk. For example, one Ethiopia‐based study suggested that girls tended to more commonly spend time at home and thereby may have had better access to meals (Shaka & Wondimagegne, 2018), pointing towards the potential importance of sociocultural practices. Additionally, there is some evidence suggesting a greater burden of infectious diseases such as malaria among boys compared with girls, with implications for increased anaemia risk in this population (Briggs et al., 2020). Importantly, whereas international and national guidelines and programmes on anaemia among adolescents have traditionally focused on girls as the more vulnerable group, our study highlights that there is an equally strong need to continue to monitor and respond to anaemia among adolescent boys, including in SSA (UNICEF, 2018; WHO, 2016).

Our observations support previous SSA‐based evidence indicating associations between measures of nutritional status and anaemia risk. In our study population, HAZ was inversely associated with anaemia risk. Similarly, stunting was associated with an increased risk of anaemia among adolescents in an Ethiopian population (Shaka & Wondimagegne, 2018). Associations between anaemia and stunting may be reflective of common exposures potentially continuing from earlier childhood that contributes to undernutrition. Previous reports have also suggested associations between dietary diversity or the consumption of specific food groups and anaemia risk, although the overall evidence is not fully consistent (Azupogo et al., 2019; Engidaw et al., 2018; Gosdin et al., 2020; Korkalo et al., 2017; Regasa & Haidar, 2019). In this study, although there was evidence of an association between dietary diversity and anaemia among adolescents in univariable models, these were not notable in multivariable models. On the other hand, poorer diet quality remained consistently associated with increased anaemia risk across adjusted models, and food insecurity was associated with higher risk of moderate or severe anaemia. These measures may have at least partly accounted for the effect of dietary diversity in this study population, alongside other included measures. Diet quality and food insecurity are reflective of modifiable nutritional circumstances; thus, further investigation of ways to improve these indices in these and similar populations may provide useful insights into avenues for anaemia reduction.

Few SSA‐based studies identified here examined the role of WASH measures or the presence of infection‐related exposures on anaemia risk in adolescents (Korkalo et al., 2017; Shaka & Wondimagegne, 2018), with no clear evidence of associations (Shaka & Wondimagegne, 2018). In the current study, we identified potential influences of hygiene‐related factors on anaemia risk, with having no handwashing stations at school being associated with approximately 30% increased risk of anaemia. Infectious agents, including intestinal helminths such as hookworm, are understood to contribute to anaemia development through inhibition of intestinal iron absorption and through promotion of chronic systemic inflammation (Baldi et al., 2020; De Benoist et al., 2008; Gyorkos & Gilbert, 2014). Previous observational studies have suggested beneficial effects of improving WASH indicators on anaemia status among children under 5 years of age, presumably through reduction of exposure to such infectious agents (Kothari et al., 2019; Yu et al., 2020). However, evidence from experimental studies has been mixed, leading some to suggest that effects of WASH measures may also be reflective of aspects of socioeconomic influences on the causal pathway to anaemia (Baldi et al., 2020; Humphrey et al., 2019; Stewart et al., 2019). Our findings suggest that these factors may also be potentially important among adolescents. Further research is warranted to more clearly elucidate such effects on anaemia among adolescents, and potential mechanisms.

Data from our study preliminarily support the potential value of strategies aiming to improve dietary and WASH indices in reducing anaemia risk in these and similar adolescent populations. Integrated, multicomponent interventions addressing such indices may help to maximize gains in the prevention of anaemia and potentially other correlated measures of malnutrition, although such interventions may require greater collaboration across sectors including WASH and social protection (UNICEF, 2020). Schools have been identified as central to such efforts, providing valuable platforms for the targeted delivery of such interventions to adolescents (McMichael, 2019; UNSCN, 2017). Supporting this, we observed an important effect of school handwashing stations, a school‐based hygiene measure, on anaemia risk, although evidence of the effects of school feeding programmes was less clear. However, given previous evidence of increased risk of poorer health measures among out‐of‐school adolescents, who constitute a notable proportion of adolescents in SSA (De Neve et al., 2020), there remains a need to devise relevant strategies that may reach individuals regardless of school‐going status (UNICEF, 2021). Additionally, we observed no differences in the patterns and magnitudes of association when examining either any anaemia or moderate to severe anaemia as an outcome and no clear differences by sex. From a programmatic perspective, these results suggest that a single approach to target anaemia at all levels of severity and for both boys and girls may be appropriate for our study populations.

This study has multiple strengths. We present evidence regarding anaemia among early adolescent boys and girls aged 10−14 in three countries across SSA, population groups from which data regarding anaemia are generally scarce. Information on a range of potential determinants at the individual, household and school levels was considered in analyses, enabling a detailed assessment of relative contributions to anaemia risk in this population. Given our sampling methods, our results may be generalizable to in‐school young adolescents in other urban and semi‐urban public school settings in our study countries, although they may not be fully representative given possible setting‐specific differences. Limitations of this study include its cross‐sectional nature, which prevents strong inferences about the temporality of observed associations. Estimates of prevalence were not weighted and are therefore crude estimates of relative burden specific to our study settings. Additionally, the overall range of risk factors and magnitudes of their associations among out‐of‐school or rural adolescents may be distinct compared with this population. More generally, a broader examination of trends in anaemia risk by urbanicity may be informative in future studies. We did not collect information related to malaria and helminth infections and geophagy, which may be relevant factors to consider in this analysis. Finally, because the stratified analyses in this study were based on relatively small sample sizes, there may have been limited statistical power to detect associations or discern differences in associations. Nonetheless, this study contributes important, up‐to‐date information regarding the status of anaemia among in‐school early adolescents in these three countries, and the need and potential approaches to address the burden of anaemia in these settings.

In all, in this study, we observed a high overall prevalence of anaemia among adolescents aged 10−14 in Ethiopia, Sudan and Tanzania, with differences in burden by country. Along with age and sex, anaemia was associated with modifiable nutrition and hygiene factors including diet quality and handwashing stations at school. Our results point towards the need to address anaemia among younger adolescents in SSA, with an equally strong focus on adolescent boys alongside girls. This study provides evidence to inform further research and relevant strategies in this area, supporting the potential use of schools as platforms for multicomponent interventions to prevent and treat anaemia among both adolescent girls and boys. Further data from across SSA will be valuable in informing the most effective strategies to address anaemia among adolescents.

## AUTHOR CONTRIBUTIONS

Wafaie W. Fawzi, Amare W. Tadesse, Huda Sherfi, Mary Mwanyika‐Sando, Roisin Drysdale and Deepika Sharma designed the study. Amare W. Tadesse, Huda Sherfi and Mary Mwanyika‐Sando performed the research, along with Wafaie W. Fawzi, Till Baernighausen, Roisin Drysdale and Deepika Sharma. Uttara Partap and Amare W. Tadesse analyzed the data and wrote the first draft of the manuscript. All authors reviewed and critically revised the manuscript, and have read and approved the final manuscript.

## CONFLICT OF INTEREST

The authors declare no conflict of interest.

## Supporting information

Supporting information.

## Data Availability

The data sets generated and analyzed during this study are available from the corresponding author upon reasonable request.
