# Supplementary material for: Burden and determinants of anaemia among in‐school young adolescents in Ethiopia, Sudan and Tanzania
Source: Matern Child Nutr. 2023 Mar 30;21(Suppl 1):e13439. doi: 10.1111/mcn.13439 (PMC12208909; doi:10.1111/mcn.13439)
Supplement: Supplementary file 1 — Supporting information. [file MCN-21-e13439-s001.docx]

| **Supplementary Table 1. Associations of individual, family and school characteristics with moderate or severe anemia among adolescents (versus no anemia) or hemoglobin in Addis Ababa (Ethiopia), Khartoum (Sudan) and Dar es Salaam (Tanzania).** | | | | |
| --- | --- | --- | --- | --- |
|  | **Moderate or severe anemia (versus no anemia)** | | **Hemoglobin** | |
|  | **N=2927** |  | **N=3553** |  |
|  | **RR (95% CI)** | ***P*** | **β (95% CI)** | ***P*** |
|  |  |  |  |  |
| **Sex of participant** |  |  |  |  |
| Male | **1.17 (1.07, 1.28)** | **<0.001** | **-0.12 (-0.14, -0.11)** | **<0.001** |
| Female | Ref |  | Ref |  |
| **Age of participant** |  |  |  |  |
| 10 – 12 years | 0.97 (0.91, 1.03) | 0.268 | **-0.18 (-0.24, -0.11)** | **<0.001** |
| 13 – 14 years | Ref |  | Ref |  |
| **Height-for-age z-score** | **0.92 (0.89, 0.95)** | **<0.001** | **0.09 (0.05, 0.12)** | **<0.001** |
| **Live with both parents** |  |  |  |  |
| No | 1.02 (0.91, 1.15) | 0.718 | 0.00 (-0.03, 0.03) | 0.960 |
| Yes | Ref |  | Ref |  |
| **Wash hands after toilet** |  |  |  |  |
| Rare/ sometimes | 0.91 (0.79, 1.04) | 0.168 | 0.05 (-0.09, 0.18) | 0.469 |
| Mostly/ Always | Ref |  | Ref |  |
| **Maternal highest education** |  |  |  |  |
| Primary or less | 1.11 (0.97, 1.28) | 0.124 | **-0.12 (-0.17, -0.06)** | **<0.001** |
| Secondary or above | Ref |  | Ref |  |
| **Dietary Diversity (MDD-W)** |  |  |  |  |
| Poor | 0.86 (0.56, 1.31) | 0.487 | -0.03 (-0.12, 0.07) | 0.601 |
| Adequate | Ref |  | Ref |  |
| **Global Diet Quality Score (GDQS)** |  |  |  |  |
| High | Ref |  | Ref |  |
| Moderate | **1.17 (1.12, 1.22)** | **<0.001** | **-0.09 (-0.17, -0.01)** | **0.022** |
| Low | **1.20 (1.08, 1.32)** | **<0.001** | -0.10 (-0.23, 0.02) | 0.105 |
| *P* for linear trend |  | **<0.001** |  | 0.231 |
| **Food insecurity (HHS)** | **1.04 (1.00, 1.07)** | **0.028** | -0.03 (-0.07, 0.01) | 0.166 |
| **School feeding** |  |  |  |  |
| No | 0.88 (0.51, 1.52) | 0.656 | 0.30 (-0.24, 0.84) | 0.275 |
| Yes | Ref |  | Ref |  |
| **School dietary guidelines** |  |  |  |  |
| No | 0.94 (0.51, 1.75) | 0.853 | 0.19 (-0.02, 0.41) | 0.073 |
| Yes | Ref |  | Ref |  |
| **School handwashing stations** |  |  |  |  |
| No | **1.33 (1.07, 1.67)** | **0.012** | -0.50 (-1.04, 0.03) | 0.066 |
| Yes | Ref |  | Ref |  |
|  |  |  |  |  |
| Estimates based on Poisson or linear regression models adjusting for all other variables displayed, and for clustering at the school and country level. 95% CI: 95% confidence interval. MDD-W: Minimum Dietary Diversity for Women of Reproductive Age. HHS: Household Hunger Scale score. | | | | |

| **Supplementary Table 2. Associations of individual, family and school characteristics with anemia among adolescents in Addis Ababa (Ethiopia), Khartoum (Sudan) and Dar es Salaam (Tanzania) - diet quality assessed using Prime Diet Quality Score.** | | | | |
| --- | --- | --- | --- | --- |
|  | **Any anemia** | | **Moderate or severe anemia^1^** | |
|  | **N=3553** |  | **N=3553** |  |
|  | **RR (95% CI)** | ***P*** | **RR (95% CI)** | ***P*** |
|  |  |  |  |  |
| **Sex of respondent** |  |  |  |  |
| Male | **1.11 (1.09, 1.14)** | **<0.001** | **1.16 (1.04, 1.29)** | **0.009** |
| Female | Ref |  | Ref |  |
| **Age of respondent** |  |  |  |  |
| 10 – 12 years | **0.91 (0.86, 0.96)** | **<0.001** | 1.06 (0.95, 1.18) | 0.334 |
| 13 – 14 years | Ref |  | Ref |  |
| **Height-for-age z-score** | **0.93 (0.90, 0.95)** | **<0.001** | **0.92 (0.90, 0.95)** | **<0.001** |
| **Live with both parents** |  |  |  |  |
| No | 1.02 (1.00, 1.05) | 0.110 | 1.02 (0.91, 1.14) | 0.757 |
| Yes | Ref |  | Ref |  |
| **Wash hands after toilet** |  |  |  |  |
| Rare/ sometimes | 0.89 (0.76, 1.05) | 0.156 | **0.91 (0.82, 1.00)** | **0.046** |
| Mostly/ Always | Ref |  | Ref |  |
| **Maternal highest education** |  |  |  |  |
| Primary or less | 1.07 (0.95, 1.20) | 0.284 | 1.08 (0.88, 1.33) | 0.442 |
| Secondary or above | Ref |  | Ref |  |
| **Dietary Diversity (MDD-W)** |  |  |  |  |
| Poor | 0.93 (0.82, 1.06) | 0.298 | 0.91 (0.55, 1.50) | 0.700 |
| Adequate | Ref |  | Ref |  |
| **Prime Diet Quality Score (PDQS)** |  |  |  |  |
| 4^th^ (highest) Quartile | Ref |  | Ref |  |
| 3^rd^ Quartile | 1.07 (0.93, 1.24) | 0.334 | 1.20 (0.88, 1.64) | 0.238 |
| 2^nd^ Quartile | **1.24 (1.04, 1.48)** | **0.018** | 1.24 (0.93, 1.66) | 0.140 |
| 1^st^ (lowest) Quartile | **1.20 (1.08, 1.33)** | **<0.001** | **1.31 (1.13, 1.52)** | **<0.001** |
| P for linear trend |  | **0.023** |  | **<0.001** |
| **Food insecurity (HHS)** | 1.00 (0.99, 1.02) | 0.557 | **1.07 (1.03, 1.11)** | **0.001** |
| **School feeding** |  |  |  |  |
| No | 1.00 (0.70, 1.42) | 0.989 | 0.84 (0.50, 1.42) | 0.514 |
| Yes | Ref |  | Ref |  |
| **School dietary guidelines** |  |  |  |  |
| No | 0.91 (0.66, 1.25) | 0.562 | 0.95 (0.53, 1.69) | 0.854 |
| Yes | Ref |  | Ref |  |
| **School handwashing stations** |  |  |  |  |
| No | **1.27 (1.21, 1.34)** | **<0.001** | 1.38 (0.94, 2.04) | 0.099 |
| Yes | Ref |  | Ref |  |
|  |  |  |  |  |
| Estimates based on Poisson regression models adjusting for all other variables displayed, and for clustering at the school and country level. ^1^Comparator: no or mild anemia. 95% CI: 95% confidence interval. MDD-W: Minimum Dietary Diversity for Women of Reproductive Age. HHS: Household Hunger Scale score. | | | | |

| **Supplementary Table 3. Associations of individual, family and school characteristics with anemia among adolescents in in Addis Ababa (Ethiopia), Khartoum (Sudan) and Dar es Salaam (Tanzania) - estimates from cumulatively adjusted models.** | | | | | | |
| --- | --- | --- | --- | --- | --- | --- |
|  | **Any anemia (N=3553)** | | **Moderate or severe anemia (versus no or mild anemia) (N=3553)** | | **Moderate or severe anemia (versus no anemia) (N=2927)** | |
|  | **RR (95% CI)** | **P** | **RR (95% CI)** | **P** | **RR (95% CI)** | **P** |
|  |  |  |  |  |  |  |
| **Sex of participant** |  |  |  |  |  |  |
| Male | 1.14 (1.14, 1.15) | <0.001 | 1.20 (1.04, 1.40) | 0.016 | 1.21 (1.06, 1.39) | 0.004 |
| Female | Ref |  | Ref |  | Ref |  |
| **Age of participant** |  |  |  |  |  |  |
| 10 – 12 years | 0.88 (0.82, 0.94) | <0.001 | 1.01 (0.92, 1.11) | 0.811 | 0.93 (0.89, 0.97) | 0.002 |
| 13 – 14 years | Ref |  | Ref |  | Ref |  |
| **Live with both parents** |  |  |  |  |  |  |
| No | 1.01 (0.94, 1.07) | 0.827 | 1.02 (0.91, 1.14) | 0.755 | 1.02 (0.91, 1.15) | 0.677 |
| Yes | Ref |  | Ref |  | Ref |  |
| **Maternal highest education** |  |  |  |  |  |  |
| Primary or less | 1.07 (0.97, 1.19) | 0.158 | 1.10 (0.82, 1.48) | 0.526 | 1.10 (0.86, 1.40) | 0.446 |
| Secondary or above | Ref |  | Ref |  | Ref |  |
| **Household asset index** |  |  |  |  |  |  |
| Lowest quintile | Ref |  | Ref |  | Ref |  |
| Low quintile | 0.95 (0.90, 1.01) | 0.083 | 0.88 (0.69, 1.11) | 0.270 | 0.91 (0.76, 1.08) | 0.283 |
| Middle quintile | 0.91 (0.78, 1.07) | 0.259 | 0.93 (0.80, 1.09) | 0.388 | 0.86 (0.73, 1.02) | 0.081 |
| High quintile | 0.92 (0.81, 1.04) | 0.170 | 0.91 (0.78, 1.07) | 0.262 | 0.87 (0.74, 1.04) | 0.119 |
| Highest quintile | 0.96 (0.81, 1.15) | 0.689 | 0.98 (0.73, 1.31) | 0.869 | 0.94 (0.71, 1.25) | 0.689 |
| **School feeding** |  |  |  |  |  |  |
| No | 1.00 (0.70, 1.43) | 0.983 | 0.84 (0.50, 1.41) | 0.508 | 0.88 (0.52, 1.50) | 0.642 |
| Yes | Ref |  | Ref |  | Ref |  |
| **School dietary guidelines** |  |  |  |  |  |  |
| No | 0.90 (0.65, 1.24) | 0.506 | 0.93 (0.52, 1.65) | 0.795 | 0.92 (0.50, 1.70) | 0.795 |
| Yes | Ref |  | Ref |  | Ref |  |
| **School handwashing stations** |  |  |  |  |  |  |
| No | 1.27 (1.21, 1.33) | <0.001 | 1.39 (0.90, 2.13) | 0.135 | 1.34 (1.02, 1.77) | 0.036 |
| Yes | Ref |  | Ref |  | Ref |  |
| **Food insecurity (HHS)** | 1.00 (0.99, 1.00) | 0.604 | 1.06 (1.01, 1.11) | 0.019 | 1.03 (0.98, 1.08) | 0.246 |
| **Dietary Diversity (MDD-W)** |  |  |  |  |  |  |
| Poor | 0.93 (0.82, 1.05) | 0.251 | 0.90 (0.55, 1.48) | 0.674 | 0.85 (0.56, 1.28) | 0.436 |
| Adequate | Ref |  | Ref |  | Ref |  |
| **Global Diet Quality Score (GDQS)** |  |  |  |  |  |  |
| High | Ref |  | Ref |  | Ref |  |
| Moderate | 1.12 (1.02, 1.24) | 0.021 | 1.14 (1.10, 1.19) | <0.001 | 1.18 (1.12, 1.24) | <0.001 |
| Low | 1.09 (0.91, 1.32) | 0.349 | 1.20 (1.14, 1.27) | <0.001 | 1.21 (1.12, 1.31) | <0.001 |
| **Wash hands after toilet** |  |  |  |  |  |  |
| Rare/ sometimes | 0.90 (0.74, 1.08) | 0.237 | 0.92 (0.82, 1.02) | 0.099 | 0.90 (0.79, 1.03) | 0.133 |
| Mostly/ Always | Ref |  | Ref |  | Ref |  |
| **Height-for-age z-score** | 0.93 (0.90, 0.96) | <0.001 | 0.92 (0.90, 0.95) | <0.001 | 0.92 (0.90, 0.94) | <0.001 |
|  |  |  |  |  |  |  |
| Estimates based on Poisson regression models adjusted as described below and for clustering at the school and country level. Model 1: adjusted for sex, age, living with both parents, maternal highest education and household asset index - estimates presented for these variables are from Model 1.  Model 2: adjusted for all variables in Model 1, plus school feeding, dietary guidelines and handwashing stations - estimates presented for these additional variables are from Model 2.  Model 3: adjusted for all variables in Model 2, plus food insecurity, dietary diversity, Global Diet Quality Score, and washing hands after going to toilet - estimates presented for these additional variables are from Model 3. Model 4: adjusted for all variables in Model 3, plus height-for-age z-score - estimate for this additional variable is from Model 4.  95% CI: 95% confidence interval. MDD-W: Minimum Dietary Diversity for Women of Reproductive Age. HHS: Household Hunger Scale score. | | | | | | |
